# Nickel-Refining Fumes Induced DNA Damage and Apoptosis of NIH/3T3 Cells via Oxidative Stress

**DOI:** 10.3390/ijerph13070629

**Published:** 2016-06-23

**Authors:** Yue Wang, Sheng-Yuan Wang, Li Jia, Lin Zhang, Jing-Chong Ba, Dan Han, Cui-Ping Yu, Yong-Hui Wu

**Affiliations:** 1 Department of Occupational Health, College of Public Health, Harbin Medical University, Harbin 150000, China; wangyue5056@163.com (Y.W.); wangshengyuan@163.com (S.-Y.W.); sleepapple@126.com (L.Z.); b_sonia@163.com (J.-C.B.); nice.hd@163.com (D.H.); cuicui790913@163.com (C.-P.Y.); 2 Department of Environmental Health, College of Public Health, Harbin Medical University, Harbin 150000, China; jiali1013@163.com

**Keywords:** nickel-refining fumes, NIH/3T3cell, DNA damage, apoptosis, oxidative stress

## Abstract

Although there have been numerous studies examining the toxicity and carcinogenicity of nickel compounds in humans and animals, its molecular mechanisms of action are not fully elucidated. In our research, NIH/3T3 cells were exposed to nickel-refining fumes at the concentrations of 0, 6.25, 12.50, 25, 50 and 100 μg/mL for 24 h. Cell viability, cell apoptosis, reactive oxygen species (ROS) level, lactate dehydrogenase (LDH) assay, the level of glutathione (GSH), activities of superoxide dismutase (SOD), catalase (CAT), and malondialdehyde (MDA) level were detected. The exposure of NIH/3T3 cells to nickel-refining fumes significantly reduced cell viability and induced cell apoptotic death in a dose-dependent manner. Nickel-refining fumes significantly increased ROS levels and induced DNA damage. Nickel-refining fumes may induce the changes in the state of ROS, which may eventually initiate oxidative stress, DNA damage and apoptosis of NIH/3T3 cells.

## 1. Introduction

As an important material in many processes of modern industry, nickel has been applied in many forms. Several million workers worldwide are exposed to airborne fumes, dusts, and mists containing nickel and its compounds [1]. Nickel compounds can enter the body through inhalation, ingestion, and dermal absorption [2,3]. The International Agency for Research on Cancer (IARC) has classified nickel as an important human carcinogen (nickel compounds Group 1, metal nickel group 2B) [4,5]. The toxicity and carcinogenicity of nickel compounds in humans and animals have been well documented [6,7,8,9,10,11]. However, the molecular mechanism involved in nickel-induced lung carcinogenesis remains unclear.

According to previous reports, several pathways were involved in nickel compounds-induced apoptosis, including JNK regulation [12], mediation of reactive oxygen species (ROS) production, inhibition of NF-kB activation, caspase-3 activation, up-regulation of Bax, and down-regulation of Bcl-2 [13,14,15]. Some researchers ascribe the pro-apoptotic properties of nickel compounds to production of ROS [16,17,18]. Oxidative stresses caused by ROS can induce rapid depolarization of inner mitochondrial membrane permeabilization (MMP) and subsequent impairment of oxidative phosphorylation [19]. This triggers either caspase-dependent or independent apoptosis. Mitochondrial apoptotic proteincytochrome c causes caspase-dependent cell death, such as caspase-3, caspase-6, caspase-9 activation, and causes oligonucleosomal DNA fragmentation during mitochondria mediated caspase-independent cell death [20,21].

Cells respond to DNA damage by activating DNA damage-inducible genes whose products contribute to cell cycle arrest and apoptosis [22]. Generation of ROS is one of the important biomarkers of cell proliferation and apoptosis [23]. Nickel induces oxidative damage, resulting in an increase of ROS production, and it is postulated that Ni-induced ROS may be involved in apoptosis. However, up to the present, it is unclear whether increasing ROS is a prerequisite for the apoptotic and genotoxic processes.

Our early research found that nickel-refining fumes might contain many metal compounds such as Ni, arsenic (As), copper (Cu), and so on [24]. Since environmental exposures normally consist of mixtures of heavy metals, it would be more meaningful to identify the pattern of cell injury and cell death induced by the nickel-refining fumes, and to explore the mechanism, rather than nickel or its compounds only. In the present study, we have hypothesized that (1) nickel-refining fumes, collected from a refining furnace workshop of China, may induce apoptosis of NIH/3T3 cells and (2) changes in the state of reactive oxygen species (ROS), which may initiate oxidative stress, DNA damage and apoptosis of NIH/3T3 cells.

## 2. Materials and Methods

### 2.1. Chemicals and Media

Nickel-refining fumes were collected from a refining furnace workshop of China. The fumes were collected in agate mortar until the number of particles, whose size was less than 5 μm, reached 99% as viewed microscopically. After 1 h of ultraviolet (UV) light radiation, the fumes were dissolved in phosphate-buffered saline (PBS, Gibcol BRL, Grand Island, NY, USA).

### 2.2. Cell Culture

NIH/3T3 cell line was obtained from Shanghai Institutes for Biological Sciences (Shanghai, China). NIH/3T3 cells were incubated in Dulbecco’s modified Eagle’s medium (DMEM, Gibcol BRL, Grand Island, NY, USA), supplemented with 10% fetal bovine serum (FBS, Hangzhou, China), 100 U/mL penicillin G, and 100 mg/mL streptomycin at 37 °C humidified atmosphere containing 5% CO_2_. All studies were performed when cells were at 50% confluence.

### 2.3. Component Analysis of Nickel-Refining Fumes

The inductively coupled plasma mass spectrometric detection method (ICP-MS, 7500ce, Agient Technologies, Santa Clara, CA, USA) was used in our study. Component analysis of nickel-refining fumes was performed following the procedure as described by Badding et al. [25]. The element concentrations in the samples could automatically be calculated by the instrument.

### 2.4. Detection of Nickel Content in Cells

Cells were seeded at a density of 5 × 10^5^ cells/well. Different concentrations of nickel-refining fumes were added to the medium. At the end of 24 h, the medium was removed, cells were washed with sterile PBS repeatedly for 2–3 times, to remove the adherent nickel compound particles in the cell surface. The cells was digested by adding 0.125% trypsin, and collected by centrifugation with 1200 r/min, 5 min. The supernatant was abandoned. Each sample was washed with 10 mL saline and the cells were suspended uniformly. At the same time, the cells were counted. Then, the cells of the samples were collected by centrifugation with 1200 r/min for 5 min. The supernatant was abandoned. The sample was dried. In addition, 500 μL of concentrated nitric acid (superior grade pure) was added to each sample tube and placed overnight. On the 2nd day after heating and digesting for 4 h at in 80 °C water bath, the samples were added with deionized water to 5.0 mL, and the content of nickel was tested by atomic absorption spectrophotometer.

### 2.5. Cellular Viability

#### 2.5.1. MTT Assay

Cell viability was determined by 3-(4,5-dimethylthiazol-2-yl)-2,5-diphenyltetrazolium bromide (MTT) assay [26,27,28]. The MTT assay assessed the mitochondrial function by measuring the ability of viable cells to reduce MTT into blue formazon product. The formazan product is impermeable to the cell membranes of viable cells accumulating inside [29]. Briefly, NIH/3T3 cells were seeded in 96-well plates at a density of 1 × 10^4^ cells/well and exposed to nickel-refining fumes for 24 h. Saline was used as negative control. After the exposure was completed, 20 μL MTT solutions was added into each well in the plate, and the 96 plates were maintained at 37 °C for 4 h. After the medium had been removed, the dye crystals were solubilized by adding 200 μL of dimethyl sulphoxide (DMSO). Absorption at 550 nm was measured using a multiplate reader (Thermo, Minneapolis, MN, USA).

#### 2.5.2. LDH Leakage Assay

Lactate dehydrogenase (LDH) is an enzyme widely present in cytosol that converts lactate to pyruvate [30]. When plasma membrane integrity is disrupted, LDH leaks into culture media and its extracellular level is elevated. LDH assay was carried out by the LDH assay kit (Jiancheng Bioengineering Institute, Nanjing, China) according to the manufacturer’s instruction, as previously described [31,32]. After exposure to nickel-refining fumes, the absorbance of solution was measured using a multiplate reader (Thermo, Minneapolis, MN, USA) at 450 nm.

### 2.6. Determination of Apoptosis

Based on the results of cytotoxicity assay, apoptotic cell death was examined [33,34,35,36]. Briefly, cells were seeded at a density of 5 × 10^5^/well. Twenty-four hours later, different concentrations of nickel-refining fumes were added to the medium for 24 h. Saline was used as negative control. After being treated with nickel-refining fumes for 24 h, NIH/3T3 cells were collected, washed twice with ice-cold PBS, and then cells were suspended in 200 μL of binding buffer and 10 μL of Annexin V-FITC for 15 min in the dark. Thereafter, 300 μL of binding buffer and 5 μL of propidium iodide were added to each sample. Finally, the cells were analyzed using flow cytometry (BD FACS Canto™ II, San Jose, CA, USA) with Cell Quest software (BD FACS Diva™, San Jose, CA, USA).

### 2.7. Electron Microscopy

NIH/3T3 cells were seeded and treated as already described. After 24 h treatment, cells were collected and centrifuged. Cells were scraped; the cell pellet was fixed in 4% glutaraldehyde for 24 h. The pellets were washed with 0.1 M cacodylate buffer, postfixed in 2% osmium tetroxide, dehydrated in acetone, and embedded in araldite. Ultrathin sections stained with uranyl acetate and lead citrate were examined using a transmission electron microscope (CM-10, Philips, Eindhoven, The Netherlands).

### 2.8. Mitochondrial ATP Measurement

Mitochondrial ATP generation was determined by the Mitochondrial ToxGlo assay (Promega, Madison, WI, USA). NIH/3T3 cells (1 × 10^4^/well) were treated with nickel-refining fumes for 24 h, and then mitochondrial ATP was measured according to the manufacturer’s instructions [37]. After measurement of the fluorescence, the cells were then incubated with ATP detection reagent. The luminescence of the cells was then measured after 5 min. The percentage of cell cytotoxicity and cellular level of ATP were expressed as a percentage of the nontreated control.

### 2.9. Detection of ROS

The production of intracellular reactive oxygen species (ROS) was measured using 2,7-dichlorofluorescin diacetate (DCFH-DA) [38,39]. We used the ROS assay kit (Beyotime Institute of Biotechnology, Nanjing, China) according to manufacture information [40]. In brief, NIH/3T3 cells were seeded in six-well plates at a density of 5 × 10^5^ cells/well and exposed to nickel-refining fumes for 24 h. Saline was used as a negative control. The positive reagent (Rosup) of reagent kit was used as positive control. Then condition medium was removed, the cells were incubated with 10 μM DCF-DA at 37 °C for 30 min and images were taken using fluorescence microscope (Axiovert 2000, Carl Zeiss, Göschwitzer, Germany).

### 2.10. Evaluation of DNA Damage

To detect cellular DNA damage as single-strand breaks, comet assay was performed as previously described [41,42,43,44]. Benzopyrene (B[a]P), purity, 99%; Sigma, St. Louis, MO, USA) was used as a positive control. Slides were viewed at 200 magnifications using fluorescent microscopy with an excitation filter of 549 nm and barrier filter of 590 nm. Comets were quantitatively analyzed using Comet Assay Software Project casp-1.2.2 (University of Wroclaw, Poland). In addition, 100 randomly selected cells from two microscope slides were analyzed and each treatment was carried out for six times.

### 2.11. Membrane Lipid Peroxidation Assay

The malondialdehyde (MDA) represents the end product of lipid peroxidation [45]. The concentration of MDA can be measured by reacting with thiobarbituric acid (TBA) to form a stable chromophoric production. The MDA levels in the cell medium were measured by using a MDA kit (Jiancheng Bioengineering Co. Ltd., Nanjing, China) according to the manufacturer’s instruction [46]. It was analyzed with thiobarbituric acid method by monitoring MDA-reactive products spectrophotometrically. The absorption was measured using a spectrophotometer (Thermo, Minneapolis, MN, USA) at 532 nm.

### 2.12. Detection of SOD

This method is based on the competition between superoxide dismutase (SOD) and tetrazolium blue for the superoxide radicals formed from the xanthineoxidase system [47]. Briefly, after exposure to nickel-refining fumes, the SOD activity was measured with a microplate spectrophotometer (Thermo, Minneapolis, MN, USA) at 550 nm, and the SOD activity was calculated according to the manufacturer’s instruction (Nanjing Jiancheng Biochemistry Co., Nanjing, China) [48].

### 2.13. Detection of GSH

Glutathione (GSH) levels in the cell extracts were determined by using a GSH kit (Jiancheng Bioengineering Co. Ltd., Nanjing, China) according to the manufacturer’s instruction [49]. Briefly, a mixture of 0.1 mL of cell extract and 0.9 mL of 5% trichloroacetic acid was centrifuged at 2300 *g* for 15 min at 4 °C. Then, 0.5 mL of the supernatant was added into 1.5 mL of 0.01% 5,5-dithio -bis-2-nitrobenzoic acid (DTNB), and the reaction was monitored at 412 nm. The amount of GSH was expressed in terms of μmol/mg protein.

### 2.14. Measurement of CAT Level

Catalatic enzymes (CAT) activity was measured with Aebi’s method [50]. Briefly, 0.1 mL supernatant was added to a quartz cuvette containing 2.95 mL of H_2_O_2_ solution (19.0 mmol/L) prepared in 0.05 M potassium phosphate buffer (pH 7.00). The change in absorbance was detected at 240 nm by using a spectrophotometer (Thermo, Minneapolis, MN, USA).

### 2.15. Statistical Analysis

Results are expressed as means and SDs. Statistical analyses were performed with one-way analysis of variance (ANOVA). Differences were considered statistically significant when *p* < 0.05.

## 3. Results and Discussion

### 3.1. The Contents of Various Elements in Nickel-Refining Fumes

**Figure 1:**
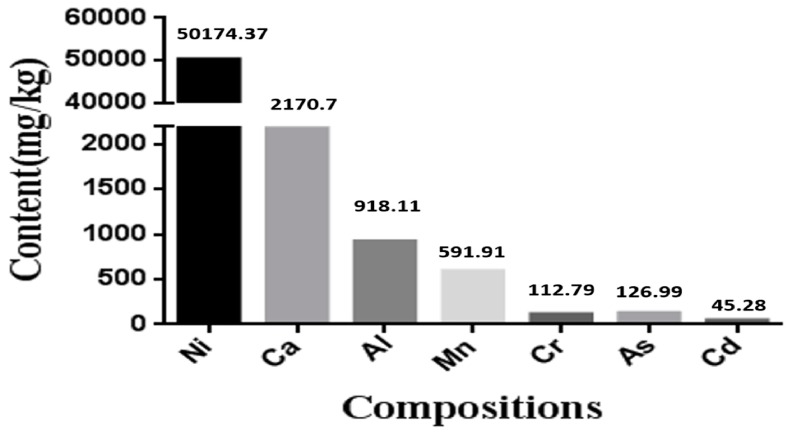
The compositions of nickel-refining fumes detected by ICP-MS. The content of nickel in nickel-refining fumes was highest, which was significantly higher than other metals.

The results of various metal contents in samples of nickel-refining fumes, detected by ICP-MS, were shown in Figure 1. Figure 1 showed that the content of nickel in nickel-refining fumes was highest, which was significantly higher than other metals, such as aluminum, arsenic, chromium, cadmium and manganese.

Nickel is used in industry for alloys, coins, batteries and electroplating. In humans, occupational exposure to acute high levels of nickel primarily leads to diseases of the lung with a high incidence of nasal and lung cancer [7]. The workers in the production process are exposed to the nickel-smelting fumes and nickel-refining fumes most often. Due to different production processes, nickel fumes with a wide variety of types produce variable toxic effects through different mechanisms [51], is the main factor to decide the carcinogenicity of the smoke, and requires further research for elucidation [52]. In our study, the nickel-refining fumes contained a variety of metals, which was similar to previous report, but the influence of nickel-refining fumes on biological systems was not known.

### 3.2. Analysis of Nickel Content in Cells

**Figure 2:**
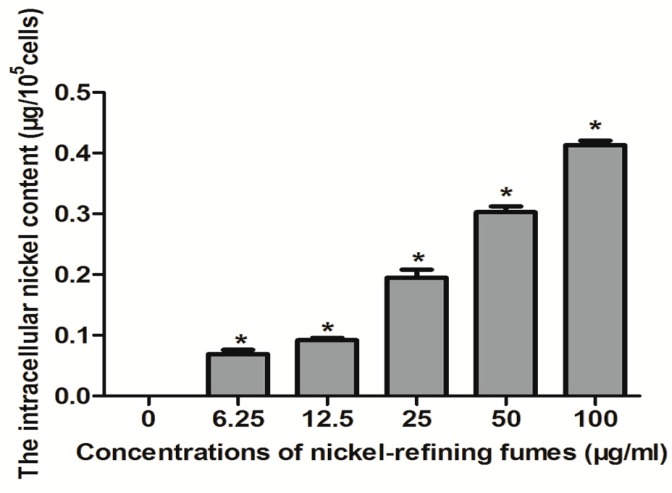
The nickel content in NIH/3T3 cells. With the increase of nickel-refining fume concentration, the content of nickel in NIH/3T3 cells gradually increased, in a dose–response relationship. Notes: Data represented are mean ± standard deviation of three identical experiments made in triplicate. * Statistically significant difference as compared to the controls (*p* < 0.05).

NIH/3T3 cells were treated with 0, 6.25, 12.50, 25, 50, 100 μg/mL of nickel-refining fumes for 24 h, and the changes of the intracellular nickel content was shown in Figure 2. Figure 2 showed that, with the increase of nickel-refining fumes concentration, the content of nickel in NIH/3T3 cells gradually increased, in a dose–response relationship. The increasing extent from 0 μg/mL to 100 μg/mL was obvious, which indicated that the particle of nickel-refining fumes could enter the cells and cause the biological effect.

A previous scientific report showed that the carcinogenicity of nickel compounds was related to its biological utilization degree. It could enter the cells by phagocytosis, calcium channel and clathrin mediated endocytosis and other pathways, which was affected by the solubility of the particles, the structure and surface charge [53]. A number of studies showed that insoluble nickel compounds entered into the cells by phagocytosis and the cytotoxicity of the nickel compounds was related to the phagocytic activity. Muñoz and others further demonstrated that the carcinogenicity of insoluble nickel compounds was proportional to the cell intake [54].

### 3.3. Inhibition of Cell Viability, Damage of Cell Membrane, Depletion of Mitochondrial ATP, Induction of Apoptosis by the Treatment of Nickel-Refining Fumes in NIH/3T3 Cells

**Figure 3:**
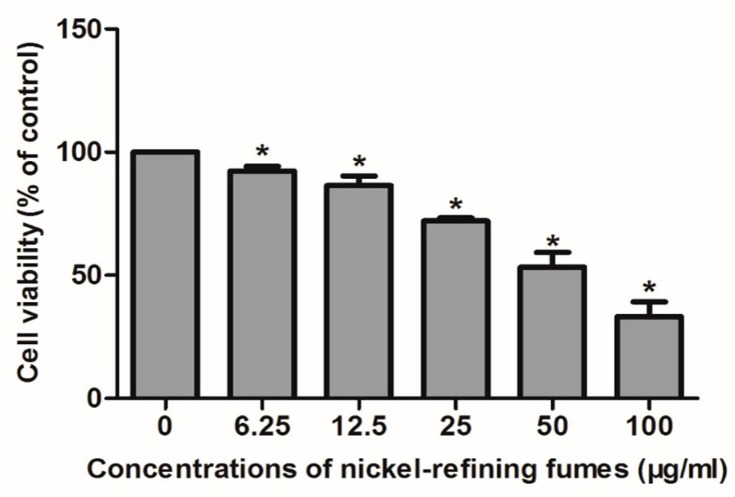
Cytotoxic effect of nickel-refining fumes to NIH/3T3 cells. NIH/3T3 cells were cultured in the absence or presence of nickel-refining fumes (0, 6.25, 12.50, 25, 50 and 100 μg/mL) for 24 h as indicated in the Materials and Methods. Cell viability was determined based on the MTT assay. Notes: Data represented are mean ± standard deviation of three identical experiments made in triplicate. * Statistically significant difference as compared with the control (*p* < 0.05).

To evaluate the effects of nickel-refining fumes on cell viability, NIH/3T3 cells were stimulated with nickel-refining fumes at the concentrations of 0, 6.25, 12.50, 25, 50 and 100 μg/mL for 24 h using the MTT assay. Nickel-refining fumes decreased the cell viability in a dose-dependent manner, as shown in Figure 3.

**Figure 4:**
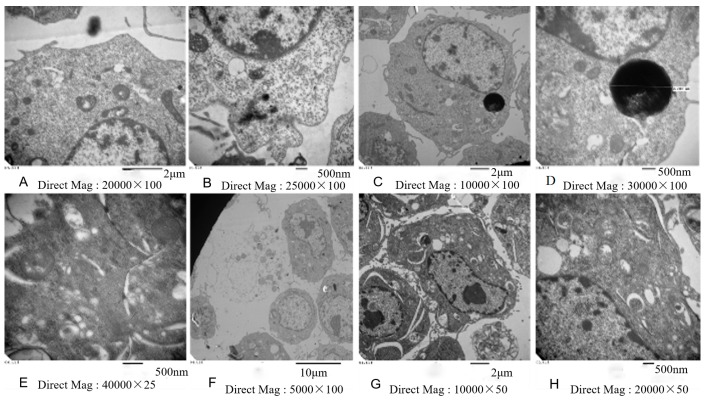
Electron microscope images of NIH/3T3 cells exposed to the nickel-refining fumes for 24 h. Note: (**A**) nickel-refining fumes particles; (**B**) the cellular uptake of nickel refining fumes particles; (**C**,**D**) nickel refining fumes particles in NIH/3T3 cells; (**E**) the formation of cell junction; (**F**) necrosis of NIH/3T3 cells; (**G**) apoptosis of NIH/3T3 cells; (**H**) vacuolar degeneration of the mitochondria.

Morphological changes of cell damage in NIH/3T3 cells were determined using transmission electron microscope. Figure 4 showed that NIH/3T3 cells could phagocytize the nickel-refining fumes particles, which had an effect of apoptosis on NIH/3T3 cells. Under electron microscope, the nucleus of apoptotic cells shrunk, chromatin agglomerated and scattered through the nucleus. The villi-like structures on cell surface disappeared. The endometrial structure degenerated. The mitochondrial vacuolar degeneration and endoplasmic reticulum expansion were observed.

**Figure 5:**
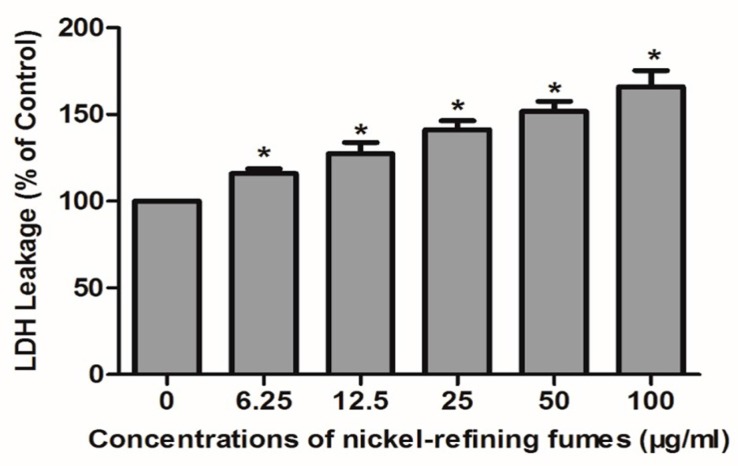
Lactate dehydrogenase (LDH) changes of the cell after the cells exposed to nickel-refining fumes for 24 h. Nickel-refining fumes induced membrane damage of NIH/3T3 cells. Note: Each data set mean value is a composite of three independent experiments with SD shown. * means statistically significant difference, compared to the controls (*p* < 0.05).

**Figure 6:**
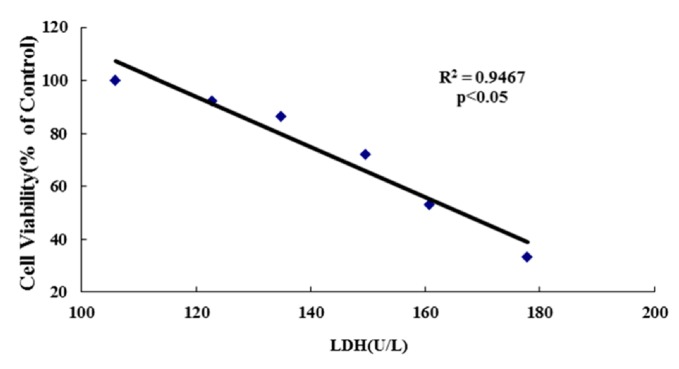
The LDH and the cell viability changes of the cell after the cells exposed to nickel-refining fumes for 24 h. Note: Significant negative correlation between the cell viability and LDH leakage after 24 h exposure to 0, 6.25, 12.50, 25.00, 50 and 100 μg/mL of nickel-refining fumes (*p* < 0.05).

The LDH assay is a cytotoxicity assay that measures membrane damage by quantifying the amount of LDH released from the cytoplasm [55,56]. Besides cell viability, cell injuries were also measured by determining LDH activities. In Figure 5, compared with control, LDH activities of the nickel-refining fumes groups were significantly increased in a dose-responsive relationship. With the rising of the exposure concentrations, LDH leakage of the cell also increased, which was consistent with MTT results, indicated that the number of cell ruptures and deaths increased. We further determined the correlation between the cell viability (MTT assay) and LDH leakage of the cells. A significant negative correlation was observed between the cell viability and LDH leakage (*p* < 0.05) (Figure 6).

**Figure 7:**
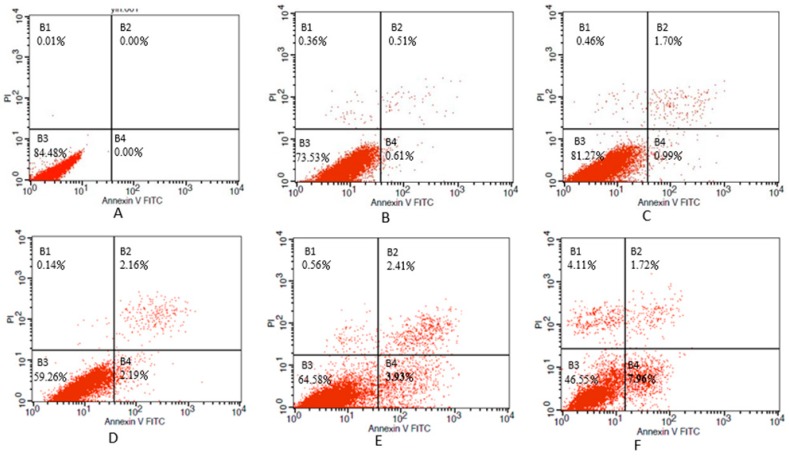
The apoptosis of NIH/3T3 cells induced by nickel refining fumes with various concentrations. Cells were treated with nickel-refining fumes at concentrations of 0, 6.25, 12.5, 25, 50 and 100 μg/mL for 24 h, then stained with Annexin V FITC and PI using flow cytometric analysis. Note: (**A**) negative control; (**B**) 6.25 μg/mL; (**C**) 12.5 μg/mL; (**D**) 25 μg/mL; (**E**) 50 μg/mL; (**F**) 100 μg/mL. B1: later apoptosis, B2: mid-term apoptosis, B3: normal, B4: early cell apoptosis.

**Figure 8:**
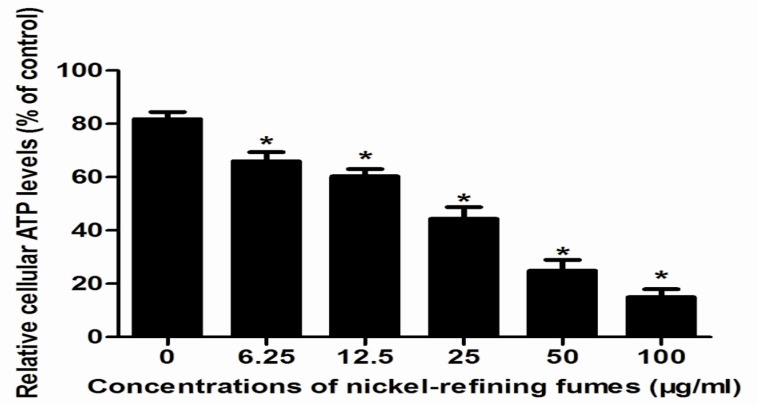
The changes of the ATP levels after the cells exposed to nickel-refining fumes for 24 h. NIH/3T3 cells were treated with nickel-refining fumes at concentrations of 0, 6.25, 2.5, 25, 50 and 100 μg/mL for 24 h. The levels of mitochondrial ATP were monitored by the ATP Determination Kit (Promega, Madison, WI, USA). * means statistically significant difference, compared to the controls (*p* < 0.05).

In order to determine whether the decrease in cell viability, observed in NIH/3T3 cells in response to nickel-refining fumes treatment was due to induction of apoptosis, flow-cytometric analysis was used to detect cells’ apoptotic death rate. The results showed that nickel-refining fumes induced cells apoptotic death in a dose-dependent manner (Figure 7). These findings suggested that NIH/3T3 cells may undergo apoptosis after exposure to nickel-refining fumes, and there was a good correlation between the extent of apoptosis and inhibition of cell growth. The effects of nickel-refining fumes on the levels of mitochondrial ATP were monitored by the ATP Determination Kit (Manufacturer, Promega, Madison, WI, USA). The results of mitochondrial ATP values showed a concentration dependent decrease in response to nickel-refining fumes treatment for 24 h, suggesting a direct role of the mitochondria in nickel-refining fumes induced apoptosis (Figure 8).

Indeed, nickel-induced apoptosis has been reported previously [57,58,59,60]. For example, Schedle demonstrated that the myeloid precursor cell line HL-60 underwent apoptosis after exposure to 1 mM NiCl_2_ [61]. Another study indicated that Chinese ovary cells showed apoptotic changes after treating them with nickel acetate [62]. Moreover, Lee and others demonstrated that undergoing apoptosis in normal rat kidney cells was induced in response to exposure to nickel (II)-induced cells [63].

In the studies of nickel induced apoptosis, the results were not consistent. In addition, 1 μmol/L of nickel could induce apoptosis of the S-100 cells by inhibiting Ca^2+^ and Mg^2+^, while 1 mmol/L nickel could not cause apoptosis in rat fibroblasts and gingival fibroblasts contact [58,64]. The research of nickel acetate on the apoptosis of Chinese hamster ovary cells found that, being exposed to more than 480 mmol/L nickel acetate for 72 h, the cell apoptosis was increased. At this concentration, exposed time reduced, the apoptosis of cells was no more than that of normal cells [61]. Similar results were found in the normal rat kidney cells treated with acetic acid [63].

### 3.4. Nickel-Refining Fumes-Induced Apoptosis Is Associated with the Generation of ROS, the Disorders in the Oxidative System, Antioxidative System and DNA Damage

**Figure 9:**
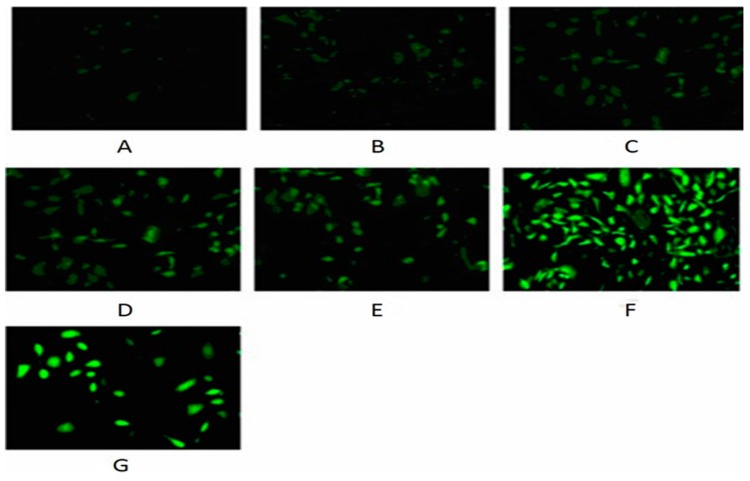
The changes of the ROS levels of NIH/3T3 cells induced by nickel-refining fumes for 24 h. Note: (**A**) negative control; (**B**) 6.25 μg/mL; (**C**) 12.5 μg/mL; (**D**) 25.00 μg/mL; (**E**) 50.00 μg/mL; (**F**) 100 μg/mL; (**G**) positive control (Rosup). NIH/3T3 cells were visualized by a fluorescent microscope (×200).

Because the generation of intracellular ROS may be related to mitochondrial dysfunction and the induction of apoptosis in various cell types, we further investigated whether nickel-refining fumes could stimulate ROS generation in NIH/3T3 cells. To accomplish this goal, we measured ROS production using the ROS-detecting fluorescent dye DCFH-DA. NIH/3T3 cells were exposed to nickel-refining fumes at the concentrations of 0, 6.25, 12.50, 25, 50 and 100 μg/mL for 24 h, the ROS levels were shown in Figure 9. The results showed that cells apoptosis had a positive correlation to ROS generation. Free Ni^2+^ acted on the mitochondria to produce a large number of ROS, which caused the imbalance of ROS content in the cells, and induced oxidative damage to cells.

Our result was also in agreement with a previous report indicating that nickel subsulfide (Ni_3_S_2_) induced ROS-mediated apoptosis in human bronchial epithelial cells (BEAS-2B) [65]. Consistent with our results, previous reports also indicated that nickel nanowires (Ni NWs) induced apoptosis through ROS generation, and that ROS induced apoptosis in HeLa cells [66]. Excess generation of ROS results in oxidative stress that mediates apoptosis. Regarding the induction of ROS by nickel-refining fumes, there is still not a clear explanation of the mechanisms.

**Figure 10:**
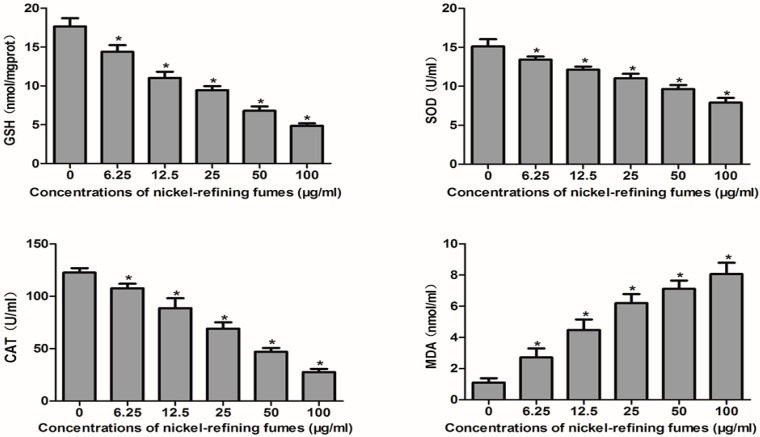
The changes of the GSH, SOD, CAT and MDA activities levels of NIH/3T3 cells, after the cells exposed to nickel-refining fumes for 24 h. Note: Each data set mean value is a composite of three independent experiments with SD shown. * Statistically significant difference as compared to the controls (*p* < 0.05).

Furthermore, to correlate these results to changes in ROS state, we evaluated the oxidative stress biomarkers including glutathione (GSH), superoxide dismutase (SOD), catalatic enzymes (CAT) and malondialdehyde (MDA) in response to nickel-refining fumes exposure. The cellular antioxidant defense system relies on the endogenous production of antioxidants, such as GSH, SOD, and CAT. However, if ROS are generated at an inappropriate time or in excessive amounts, or if antioxidant defenses are overwhelmed, negative consequences of oxidative stress may occur. Apoptotic cell death was irreversible when the antioxidant defense system was totally destroyed. We demonstrated that GSH, SOD, CAT and MDA activities levels of NIH/3T3 cells in the nickel-refining fumes groups were significantly decreased as compared to the control, which caused elevated oxidative stress (Figure 10). We also found the production of MDA and LDH increased, which were indicators of lipid peroxidation and membrane damage, respectively. Here, we could confirm that nickel-refining fumes caused imbalance of oxidant system and antioxidant defense system, increased the formation of ROS that could lead to oxidative stress, DNA damage and ultimately cell apoptosis in NIH/3T3 cells. A previous scientific report showed that NiSO_4_ can decrease the GSH levels and activities of SOD [67]. Ahamed suggests that nickel nanoparticle (NiNPs) induces oxidative damage, which decreases glutathione (GSH) and induces ROS and lipid peroxidation (LPO) in human lung epithelial A549 cells [68].

### 3.5. Nickel-Refining Fumes-Induced DNA Damage

**Figure 11:**
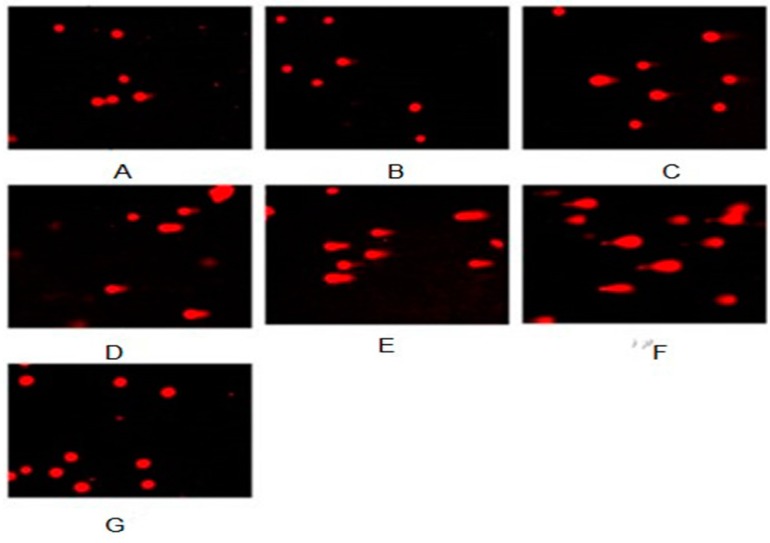
Nickel-refining fumes induced DNA damage. A significant increase in the mean of the arbitrary units was observed following exposure to increasing concentrations of nickel-refining fumes. Note: (**A**) 6.25 μg/mL; (**B**) 12.5 μg/mL; (**C**) 25.00 μg/mL; (**D**) 50.00 μg/mL; (**E**) 100 μg/mL; (**F**). positive control (B[a]P); (**G**) negative control.

It is well known that various toxic agents can induce DNA damage. DNA fragmentation is a biochemical hallmark of apoptosis. Induction of apoptosis has been recognized as a possible outcome of DNA damage for more than 35 years [69]. Single cell gel electrophoresis (SCGE) or comet assay is a simple, rapid, and sensitive technique for measuring DNA damage. DNA damage in NIH/3T3 cells exposed to nickel-refining fumes was estimated as arbitrary units by the comet assay. As shown in Figure 11 and Table 1, a significant increase in the mean of the arbitrary units was observed following exposure to increased concentrations of nickel-refining fumes. The arbitrary unit was selected for evaluation because it was considered a sensitive measure of DNA damage, based on the length of migration and the amount of DNA in the tail [42].

Oxidative stress and DNA damage are two well-documented cellular changes brought about by Ni^2+^ that contribute to toxicity and carcinogenesis [70]. It has also been established that apoptotic cell death can be triggered by extrinsic signals such as death ligands and by intrinsic signals such as DNA damage. Next, we further used comet assay to examine DNA damage response of nickel-refining fumes to explore the mechanism of apoptotic cell death induced by nickel-refining fumes. Under the fluorescence microscope, NIH/3T3 cells in the negative control group were round with no comet formation. Comet tails were observed in cells treated by nickel-refining fumes with different concentrations. With the increase of nickel-refining fumes’ concentration, the comet cell rate, tail moment and DNA content of the tail increased. Compared with the negative control group, the comet cell rate, tail moment and DNA content of the tail were significantly higher (*p* < 0.05), which indicated that nickel-refining fumes could induce DNA damage.

In aerobic cells, ROS was generated as a by-product of normal mitochondrial activity. If not properly controlled, ROS could cause severe damage to cellular macromolecules, especially the DNA. Intracellular generation of ROS was a crucial factor not only in apoptotic pathways but also in DNA damage including a multitude of oxidized base lesions, a basic site, single and double-strand breaks, and many other cellular processes which could be cytotoxic, genotoxic or mutagenic [71]. Here, we could confirm that nickel-refining fumes caused an imbalance of oxidant system and antioxidant defense system, increased the formation of ROS that might lead to oxidative stress, cyto/genotoxicity and DNA damage, and ultimately cell apoptosis in NIH/3T3 cells.

## 4. Conclusions

The present in vitro study demonstrates that nickel-refining fumes exposure gradually decreases the viability of NIH/3T3 cells, and increases lipid hydroperoxide levels resulting from reactive oxygen species formation, induces DNA damage and triggers apoptosis of NIH/3T3 cells. In summary, these findings suggest that oxidative stress might play a role in nickel refining fumes-induced cyto/genotoxicity and apoptosis of NIH/3T3 cells. This study, therefore, provides insight into the mechanism, underlying nickel refining fumes-induced toxicity and apoptosis of NIH/3T3 cells.

## Figures and Tables

**Table 1:** DNA damage of NIH/3T3 cells induced by nickel-refining fumes (*n* = 600).

Groups	Tail DNA Contents (%)	Comet Cell Rate (%)	Tail Length(x ± s, µm)
negative control	5.480 ± 0.138	3.50	8.721 ± 0.350
6.25	6.334 ± 0.247	6.17	9.041 ± 0.326
12.50	8.010 ± 0.316 *	15.33	10.509 ± 0.698
25.00	10.031 ± 0.469 *	32.83	15.175 ± 0.712 *
50.00	13.883 ± 0.683 *	51.83	17.624 ± 0.659 *
100.0	16.964 ± 0.572 *	66.50	24.974 ± 0.562 *
B[a]P	24.232 ± 0.764	96.17	34.721 ± 0.650

Nickel-refining fumes induced DNA damage. Comets were quantitatively analyzed using Comet Assay Software. 100 randomly selected cells from two microscope slides were analyzed and each treatment was carried out for six times. Note: * compared with control, *p* < 0.05.
